# Osteopathy Bill Defeated

**Published:** 1905-05

**Authors:** 


### Osteopathy Bill Defeated.

AS THESE pages are being printed a press despatch is being sent out which reads as follows:

ALBANY, April 25.—The senate today by two votes failed to pass Senator Davis's bill providing for the state regulation of the practice of osteopathy, after a long debate, in which the introducer and Senator Grady led the defense and Senators White and Marks the attack.

Senator Grady recalled the day when homeopaths were called lunatics and said that the osteopaths asked nothing more than the state protection accorded barbers, blacksmiths, embalmers and chiropodists.

"All a man needs for the practice of osteopathy," said Senator Marks, "is a good set of muscles and a smooth tongue. You can get just as valuable treatment in a Turkish bath."

Senator Hinman argued that the proposed law would be an improvment, because at present any man could set up as an osteopath; but Senator White replied that it would be dangerous for the state to dignify by its supervision any but the recognised schools of medicine.

Senator Malby joined the opposition while Senator Brackett spoke for the bill.

The bill was lost by a non-partisan vote of ayes 24; nays 19. A legal majority requires 26 votes.

Senator Davis will make another effort to pass the bill later.

THE *London Chronicle* tells this story: "She was a dreadful wreck when they brought her in to Saint Bartholomew's Hospital last evening. The youthful surgeon worked away upon her face with sponges and plaster and cotton wool, wondering aloud how she could have got into such a state. Clearly the woman had something to say,—but she could not say it. When the surgeon had made a job of it, he gently lifted the woman over an arm, and asked, 'How did it happen?' She raised a fist to shake over his shoulder, and cried furiously, ''E done it! 'E done it!—'im.' Turning his head, the surgeon could see the man, who had been standing just under the flaring gas jet, and watching the proceedings with the interest of a landed proprietor at a building operation. 'The brute!' muttered the surgeon, as he shifted the woman to her feet. She turned on him. 'Brute!' she shrieked through her bandage. 'You call 'im brute? And after he brought me all the way 'ere in his arms, Gawd bless 'im!' "—*The Tribune.*

---

UNDER Topics of Public Interest, elsewhere in the JOURNAL, we publish an article on "Paper milk bottles." Everyone interested in obtaining pure milk will find it entertaining. Dr. Ernest Wende, during his administration of the health department of Buffalo, abolished the long tube nursing bottle because of its unsanitary condition. If, acting on similar lines, the glass milk bottle should be put out of service, it would be well.

---

SPINSTERS and others addicted to floriculture and the tending of house plants should be instructed that many flowers of innocent reputation heretofore, are now being scientifically arraigned as bearers of various kinds of infection. The China primrose is a great sinner in this way, and the *Bordeaux Medical Journal* presents a considerable number of cases of dermatitis caused by it. The chrysanthemum also comes in for a share of the arraignment, as also do several varieties of rhus, which are extensively used in this country as house plants, on account of their fine foliage. Some people, the article says, cannot even touch them without being attacked by eruptions. As the domestic parrot commonly decorates the domestic conservatory, flooding it with unrestrained and generally irrelevant conversation, it may be added that this bird is at times a disseminator of infectious disease also. The skin of those who handle and caress it breaks out into eruptions, the malady being diagnosticated as psittacosis, which sometimes takes on malignant symptoms, and the patient dies. Lovers of flowers and parrots will do well to take heed of these scientific declarations concerning the danger which lurks in them, though it ought to have been found out long ago if it had been as serious as it is now affirmed to be.—*The Tribune,* April 20, 1905.

---

A LETTER FROM HOME.

Dear Jim: The crops is doing well,
The calf is big enough to sell;
I've traded off the brindle cow,
And we ain't got but one just now.
The hosses all is fat and sleek,
Except that Bob is ruther weak,
But that ain't nothing very queer;
We've had him nigh on twenty year.
I think I'll put the bottom field
In corn and oats; it oughter yield
A heavy crop; the land is rich,
And just the thing for oats and sich.
There ain't no news to speak of, Jim;
Miss Susie Jones is just as trim
As when you saw her in the fall.
The folks is well; I guess that's all—
But stop! I 'most forgot 'bout dad.
I 'xpect the news'll make you sad.
You know that dad was getting old;
Just sixty years had o'er him rolled,
And so, I must regret to say,
*We chloroformed poor dad today.*
And that is all the news until
I write again.
Your brother,
BILL.

—*Maryland Med. Jour.*

---

THE President, in promptly reorganising the Isthmian canal commission, has again shown his great executive capacity as well as his grasp of public questions of supreme magnitude. It is apparent that the report of Dr. Charles A. L. Reed on the sanitary conditions of the Isthmus had a beneficial effect in clearing the way for the action of the President and the Secretary of War.

Dr. Reed rendered a most valuable public service when, without circumlocution or indirection, he proceeded to tell the facts which he personally observed on the ground, and to put them into such unmistakable English as to attract the attention of the whole country. The government wheels would not slip their cogs if other inspectors would follow the pace set by Dr. Reed.

The report was published in the JOURNAL for April, 1905, and those who have not read it will find it of absorbing interest.

---

DR. FRANKLIN STEWART TEMPLE, who was associated with Antonius, the Boy Wonder, as a fake healer, will have to pay the fine of $100 and costs imposed upon him by Justice Kruse for failing to register here as a licensed physician.

A week ago Monday the aldermen voted to reduce the fine to $50. The councilmen refused to concur in that action. Yesterday the aldermen, after refusing in all seriousness to concur in the action of the upper house, promptly faced the other way.—*Buffalo Express,* April 25, 1905.

---

AN OSTEOPATHIC bill has been passed by the legislature of Pennsylvania and is now in the hands of the governor. The Philadelphia County Medical Society at a recent meeting appointed a committee, composed of Drs. L. Webster Fox, J. Madison Taylor, Hobart A. Hare, Alfred Stengel, Henry Beates, Jr., and Charles H. Frazier, to place before the governor the reasons why the bill should not become a law. It ought not to be difficult for the executive, after listening to this intelligent and learned body of men, to reach a proper conclusion and we feel sure that a veto of the bill will follow.

---

THE supreme court of Ohio has handed down a decision which embraces the following: The giving of christian science treatment, for a fee, for the cure of disease is practising medicine within the meaning of the statute regulating such practice in this state. Legislation prohibiting any one from treating a disease for a fee, excepting such persons as have qualifications, is a valid exercise of the police power of the state, and is constitutional.

Judge Davis, of Philadelphia, in a recent law case said: The practice of medicine consists in the offering of service and assuming the responsibilities of treating disease, deformities, and injuries, no matter by what means this is professed to be done.

In view of these judicial opinions it should not be difficult to obtain a definition of the "practice of medicine" propounded by the courts of New York. The district attorney who will present a case for judicial opinion involving this point, will be entitled to the gratitude of the medical profession.

